# The Impact of COVID-19 Pandemic on Clinical Research in China: Challenges and Progress

**DOI:** 10.3389/fmed.2020.00523

**Published:** 2020-09-11

**Authors:** Yuan Yang, Wen Li, Ling Zhang, Qinge Zhang, Teris Cheung, Brian J. Hall, Yu-Tao Xiang

**Affiliations:** ^1^Faculty of Health Sciences, Institute of Translational Medicine, University of Macau, Macao, China; ^2^Southern Medical University Nanfang Hospital, Guangdong, China; ^3^The National Clinical Research Center for Mental Disorders & Beijing Key Laboratory of Mental Disorders, Beijing Anding Hospital & The Advanced Innovation Center for Human Brain Protection, Capital Medical University, Beijing, China; ^4^School of Nursing, Hong Kong Polytechnic University, Hong Kong, China; ^5^New York University (Shanghai), Shanghai, China

**Keywords:** COVID-19, China, clinical, research, progress

## Introduction

The Novel Coronavirus Disease 2019 (COVID-19 hereafter) was first reported in Wuhan, Hubei province, China at the end of 2019, and then was found in more than 200 countries worldwide. As of April 2020, the COVID-19 outbreak has been well-contained in China. It is beyond doubt that the COVID-19 outbreak has greatly transformed clinical research in China, and this transformation is of interest to the international community. Hence, we believe that a balanced and comprehensive overview describing the impact of COVID-19 on clinical research, the challenges researchers are facing, and an update on the progress of clinical research in China is warranted.

### Research of Traditional Chinese Medicine During the COVID-19 Pandemic

Unlike other countries affected by COVID-19, traditional Chinese medicine (TCM) in China was widely prescribed in the prevention and treatment of COVID-19 among patients in clinical settings. A recent report released by the Information Office of the State Council, entitled “China Action Against the Novel Coronavirus Disease 2019,” systematically introduced the functional role of TCM in the prevention and treatment of COVID-19 infections (1). In the past few months, around 92% of COVID-19 patients have been treated with TCM, with response rates reaching 90% in Hubei province, China (1). Some studies purported that certain TCM, such as Lianhuaqingwen (LH), a repurposed marketed product composed of 13 herbs, could effectively ameliorate COVID-19–related symptoms, such as fever, cough, and fatigue, and shorten the course of the disease (2). According to the 7th edition of the “Diagnosis and Treatment Protocol for Coronavirus Pneumonia” issued by the National Health Commission and the State Administration of Traditional Chinese Medicine, LH capsules were endorsed for the treatment of COVID-19 (3).

Ever since the outbreak of COVID-19, several preliminary studies have examined certain TCM with potential antiviral effects. For example, an open-label randomized controlled trial (RCT) found that LH capsules showed therapeutic effects on COVID-19 by improving the recovery rate of certain symptoms (especially mild symptoms), shortening the course of the illness, and improving chest radiologic abnormalities (2). An *in vitro* study found that LH significantly inhibits Severe Acute Respiratory Syndrome Coronavirus 2 (SARS-COV-2) replication, affects virus morphology, and exerts anti-inflammatory activity (4). These findings suggest that TCM provides antivirus effects along with symptomatic relief, and should be adopted as a novel supportive strategy in treating COVID-19. However, these findings are tentative due to methodological deficiencies, such as the lack of a double-blind design.

It is noteworthy that the recommendations on use of TCM for COVID-19 were mainly based on expert consensus, and solid evidence based on stringent RCTs demonstrating the safety and efficacy of TCM for COVID-19 is still insufficient. TCM emphasizes individualized treatment and syndrome differentiation even for patients with the same disease and severity (5). This individualization may hinder large-scale RCTs that examine the efficacy and safety of many TCM treatment approaches to COVID-19. In addition, the findings that 90% of COVID-19 patients improved from TCM treatments were mainly based on observational studies, but not RCTs. Therefore, considering that evidence of safety and effectiveness is indispensable, large-scale and double-blind RCTs on TCMs for COVID-19 are still warranted to meet the requirements of evidence-based medicine.

### Clinical Research in China During the COVID-19 Pandemic

A recent review found that until March 26, 2020, a total of 681 COVID-19–related clinical trials have been registered on ClinicalTrials.gov and Chinese Clinical Trial Registry (ChiCTR), of which 481 had been conducted in China (6). Most of these registered studies focused on TCM, antiviral therapy, stem cell therapy, and plasma treatment; among these studies, 190 were RCTs (6, 7). On the one hand, the large number of registered trials reflect a heightened clinical research awareness and improved academic ability in China. On the other hand, however, many registered trials have obvious methodological limitations in terms of experimental design, biostatistics, data management, and preliminary data collection. For example, more than half of the registered clinical trials in China had a sample size of <100, whereas more than 30% of the clinical trials conducted in the USA, Italy, and France have a sample size of more than 500 (6). More importantly, a significant fraction of these registered clinical trials in China have failed to provide key information, such as treatment plan, drug dosage, study duration, or inclusion/exclusion criteria (7).

Because of the large number of registered clinical trials, alongside the implementation of quarantine measures in many areas, participant recruitment and follow-up assessments became immensely difficult in China (7). To ensure the quality of clinical trials and avoid wastage of resources, the Ministry of Science and Technology of China has thus enacted a strict supervision regulation, including a compulsory registration system and ethics examination procedure (7).

Since the COVID-19 outbreak, a huge volume of relevant publications drafted by researchers in China have been published, particularly in international peer-reviewed journals. For example, in a search for Severe Acute Respiratory Syndrome (SARS) and COVID-19–related publications in both PubMed and the China National Knowledge Infrastructure (CNKI) databases using the following search terms: “coronavir^*^,” “severe acute respiratory syndrome,” “SARS,” “novel coronavir^*^,” “COVID,” “COVID-19,” “China,” and “Chinese,” a total of 1,215 SARS-relevant English-language and 19,834 Chinese-language publications were retrieved from 2003 to 2004 (2 years). In contrast, during the period of January 1, 2020 to June 15, 2020 (only 5.5 months), 22,730 English and 17,098 Chinese COVID-19-related articles were retrieved. Specifically, only 58 SARS-related studies conducted in China were published on top international journals including the *Lancet, JAMA, British Medical Journal* (BMJ), and *New England Journal of Medicine* (NEJM) during the SARS outbreak. Nevertheless, during the period of January 1, 2020 to June 15, 2020, the corresponding figure of COVID-19–related publication has reached 417. This remarkable increase in the number of research outputs in China may be partly due to the growing attention to clinical research and the increasing academic communication with international community in the past two decades.

## Discussion

Despite the increasing number of publications during the COVID-19 outbreak in China, there are still some challenges to be overcome. First, standardized, sophisticated, and well-designed RCTs with large sample size are rare, which leads to insufficient empirical evidence affirming the safety and efficacy of TCM treatments for COVID-19. Second, most studies are cross-sectional and therefore, casual inferences are limited. Third, many of the clinical studies conducted in China are published in English journals, which compromises the dissemination of the study findings to frontline health professionals in China due to language barrier. Although some journals provide both English and Chinese versions of COVID-19–related articles, most other journals did not follow suit. In view of this, the Ministry of Science and Technology of China strongly encourage local researchers to publish their studies in Chinese journals instead of English journals since the end of January 2020. The Ministry of Education has also implemented new regulations that English articles are no longer listed as a pre-requisite requirement for professional/academic promotion (8). This state-sponsored push toward publications in Chinese may be a two-edge sword. On the one hand, this policy may make high-quality publications become more accessible to frontline health professionals who have difficulty reading English in China, but on the other hand, it will reduce the likelihood that they engage with a wider scientific community, thereby potentially limiting the rigor and relevance of these studies relative to those reported in international journals.

In conclusion, the Chinese government and health sector have worked hard to make significant progress in improving clinical research. Consequently, clinical research has substantially advanced and flourished in China especially during the COVID-19 pandemic, although certain challenges are yet to be overcome. Lessons learned through the development of clinical research in China may be useful to address similar challenges in future pandemics.

## Author Contributions

YY, WL, LZ, and QZ: article draft. TC, BH, and Y-TX: article revision. All authors contributed to the article and approved the submitted version.

## Conflict of Interest

The authors declare that the research was conducted in the absence of any commercial or financial relationships that could be construed as a potential conflict of interest.
